# PLACE AND AGING WITH DEMENTIA: NEW ASSESSMENTS FOR PERSON-ENVIRONMENT PROCESSES

**DOI:** 10.1093/geroni/igae098.0537

**Published:** 2024-12-31

**Authors:** Frank Oswald, Habib Chaudhury, Hans-Werner Wahl

**Affiliations:** Chair; Co-Chair; Discussant

## Abstract

There has been growth in research addressing stability and change in the relationship between the physical environment and older persons, particularly people living with dementia. However, differentiated assessments to examine or measure person-environment relationships are limited. Additionally, there is a lack of resources to inform development of physical environments supportive of people living with dementia. This symposium addresses creative measures, and application strategies to advance understanding of the role of place for people living with dementia. First, Florack and colleagues present a three-step modification of the Meaning of Home Questionnaire (MoH) for use with people living with dementia by means of simplification and addition of specific items, an initial evaluation of psychometric characteristics, and the development of a final version and its validation. Second, Niedoba and Oswald present findings from walking interviews with people living with dementia, highlighting that these interviews allow to observe and discuss everyday life person-environment processes and provide a comfortable atmosphere promoting personal reflections on the experience of living with dementia. Third, Margot-Cattin and Gaber provide evidence from the Participation in ACTivities and Places OUTside Home (ACT-OUT) questionnaire based on a transactional perspective of the person-environment relationship, showing that people with dementia experience a higher rate of abandonment of places than people without dementia. Four, Chaudhury and colleagues introduce three knowledge mobilization resources that can inform and engage with various stakeholders, including municipalities, community-based organizations, advocacy groups, planning and design professionals, persons living with dementia and care partners. Finally, Wahl serves as the session’s discussant.

